# T Cell Immunity to the Alkyl Hydroperoxide Reductase of *Burkholderia pseudomallei*: A Correlate of Disease Outcome in Acute Melioidosis

**DOI:** 10.4049/jimmunol.1402862

**Published:** 2015-04-10

**Authors:** Catherine Reynolds, Amélie Goudet, Kemajittra Jenjaroen, Manutsanun Sumonwiriya, Darawan Rinchai, Julie Musson, Saskia Overbeek, Julia Makinde, Kathryn Quigley, Jiten Manji, Natasha Spink, Pagnarith Yos, Vanaporn Wuthiekanun, Gregory Bancroft, John Robinson, Ganjana Lertmemongkolchai, Susanna Dunachie, Bernard Maillere, Matthew Holden, Daniel Altmann, Rosemary Boyton

**Affiliations:** * Lung Immunology Group, Section of Infectious Diseases and Immunity, Department of Medicine, Imperial College London, London W12 ONN, United Kingdom;; † CEA, Institut de Biologie et de Technologies de Saclay, Labex Laboratoire de Recherche sur le Médicament et l'Innovation Thérapeutique and Institut de Recherche Vaccinale, Service d'Ingénierie Moléculaire des Protéines, 91191 Gif sur Yvette, France;; ‡ Mahidol Oxford Tropical Medicine Research Unit, Faculty of Tropical Medicine, Mahidol University, Bangkok 10400, Thailand;; § Centre for Research and Development of Medical Diagnostic Laboratories, Faculty of Associated Medical Sciences, Khon Kaen University, Khon Kaen 40000, Thailand;; ¶ Institute of Cellular Medicine, Newcastle University, Newcastle upon Tyne NE2 4HH, United Kingdom;; ‖ Department of Immunology and Infection, Faculty of Infectious and Tropical Diseases, London School of Hygiene & Tropical Medicine, London WC1E 7HT, United Kingdom;; # Cambodia Oxford Medical Research Unit, Angkor Hospital for Children, Siem Reap, Cambodia;; ** Centre for Tropical Medicine, University of Oxford, Oxford OX3 7FZ, United Kingdom; and; †† School of Medicine, University of St Andrews, St Andrews KY16 9TF, United Kingdom

## Abstract

There is an urgent need for a better understanding of adaptive immunity to *Burkholderia pseudomallei*, the causative agent of melioidosis that is frequently associated with sepsis or death in patients in Southeast Asia and Northern Australia. The imperative to identify vaccine targets is driven both by the public health agenda in these regions and biological threat concerns. In several intracellular bacterial pathogens, alkyl hydroperoxidase reductases are upregulated as part of the response to host oxidative stress, and they can stimulate strong adaptive immunity. We show that alkyl hydroperoxidase reductase (AhpC) of *B. pseudomallei* is strongly immunogenic for T cells of ‘humanized’ HLA transgenic mice and seropositive human donors. Some T cell epitopes, such as p6, are able to bind diverse HLA class II heterodimers and stimulate strong T cell immunity in mice and humans. Importantly, patients with acute melioidosis who survive infection show stronger T cell responses to AhpC relative to those who do not. Although the sequence of AhpC is virtually invariant among global *B. pseudomallei* clinical isolates, a Cambodian isolate varies only in C-terminal truncation of the p6 T cell epitope, raising the possibility of selection by host immunity. This variant peptide is virtually unable to stimulate T cell immunity. For an infection in which there has been debate about centrality of T cell immunity in defense, these observations support a role for T cell immunity to AhpC in disease protection.

## Introduction

Melioidosis is increasingly considered a major and emerging public health risk in several countries in Southeast Asia, including Thailand, Cambodia, and Laos, as well as the northern territories of Australia (1–3). The disease, caused by environmental exposure to the gram-negative bacterium *Burkholderia pseudomallei*, is associated with heterogeneous presentations, from asymptomatic seroconversion to localized tissue infections, lung and brain abscesses, pneumonia, sepsis, and death. Diabetes is a major risk factor for severe, symptomatic disease (4). There are concerns that the disease is underreported in regions of similar latitude in Asia, African, and South America and that this, coupled with the growing prevalence of diabetes in these areas, may lead to a considerable increase in the melioidosis disease burden, with an at-risk population of >1 billion people (5). Furthermore, there is considerable sequence conservation between this bacterium and the related *Burkholderia* species, *Burkholderia cepacia* complex, that threaten the health of cystic fibrosis patients, making the question of T cell immunity to *Burkholderia* Ags one of relevance also in this context (6, 7).

The immunologic correlates of protection and susceptibility in this infection are poorly understood. Studies in murine models suggest that T cell–mediated IFN-γ responsiveness is important for protection (8). In humans, the immune correlates of disease susceptibility are somewhat unclear (9). The fact that diabetes is a risk factor associated with poor survival suggests a predisposing role of immune dysregulation, although the precise mechanisms have not been established (10).

Nearly 200 *B. pseudomallei* genomes of isolates derived from several countries have been sequenced. Genome size is variable, with an average size of 7.15 Mb. Faced with the challenge of establishing immunogenicity and immunodominance in a genome of this size, Felgner et al. (11) reported a protein array approach to serologic screening. A *B. pseudomallei* proteome array was expressed on chips carrying 1205 proteins predicted to be surface-expressed by PSORT, as well as components of the three different type III systems, components of flagella, proteins known to be immunoreactive from two-dimensional gels and 672 other proteins selected at random. These 1205 proteins were screened against 747 individual patient sera from 10 patient groups, including melioidosis patients and healthy seropositive donors from Thailand and Singapore; 108 Ags were described as immunodominant, and 31 were serodiagnostic for melioidosis. *Burkholderia pseudomallei* is an intracellular pathogen, making it highly likely that an effective vaccine may need to be able to activate both T cell and B cell immunity. Both a translational interest in vaccine targets and a basic interest in how the host recognizes what is essentially an intracellular pathogen of APCs highlights a need to understand T cell immunity in melioidosis.

One of the serodominant *B. pseudomallei* Ags identified in the protein array study was the alkyl hydroperoxide reductase (AhpC) BPSL2096. It is a member of the highly conserved family of peroxiredoxins, first described in yeast in the mid-1990s, with respect to the ability to protect against oxidation by reducing hydroperoxides in a thiol environment (12). In eukaryotic cells, family members are implicated in regulating resting levels of cellular peroxide to calibrate the difference between damaging oxidative stress and a beneficial role in signal transduction (13). For example, peroxiredoxin-2 is one of the most abundant RBC proteins after hemoglobin (14). The hydroperoxide reductases are represented in most bacterial species, where production of antioxidant enzymes is considered a key defense against oxidative DNA damage (15). Biofilm-derived *Burkholderia cenocepacia* are resistant to high concentrations of reactive oxygen species, attributable to transcriptional upregulation of AhpC (>40-fold) and other genes involved in the response to reactive oxygen species (16).

A key issue is the effect of human genetic heterogeneity, especially of HLA alleles, on the immune repertoire and the disease outcomes (17). In this study, we investigated whether there are shared patterns of immune response that transcend these genetic differences. The hope is that answers to these questions will facilitate novel vaccine strategies.

## Materials and Methods

### Ethics statement

Mouse experiments were performed within U.K. Home Office legislation under the terms of the Project License PPL 70/7708 and 70/8110 granted for this work under the “Animals (Scientific Procedures) Act 1986.” Local ethical review and formal approval had also been obtained through the Imperial College Ethical Review Process Committee. Leukocytes from exposed seropositive donors to the blood bank in Khon Kaen, Thailand, were collected through the Blood Transfusion Center, Khon Kaen Hospital (Khon Kaen, Thailand). Ethical permission was obtained from Ethical Khon Kaen University research no. HE470506. Blood was collected from 41 patients with cultured-confirmed melioidosis at Sappasitthiprasong Hospital, Ubon Ratchathani, at a median of 5 d after admission to hospital (interquartile range 4–6 d). Ethical approval was obtained from the Ethics Committee of the University of Mahidol Faculty of Tropical Medicine (submission number TMEC 12-014), the Ethics Committee of Sappasitthiprasong Hospital, Ubon Ratchathani (reference 018/2555), and the Oxford Tropical Research Ethics Committee (reference 64-11). The study was conducted according to the principles of the Declaration of Helsinki (2008) and the International Conference on Harmonization Good Clinical Practice guidelines. Written informed consent was obtained for all patients enrolled in the study.

### BPSL 2096 protein and peptides

The AhpC (BPSL2096) sequence (accession no. YP_108693.1) of *B. pseudomallei* was cloned, recombinant AhpC expressed in *Escherichia coli*, and purified (Biomatik, Cambridge, ON, Canada). Synthetic peptides of 20 aa in length and overlapping by 10 aa based on the sequence of *B. pseudomallei* strain K96243 were synthesized by GL Biochem (Shanghai, China) (Table I).

### HLA-peptide binding assays

HLA-DR and -DQ heterodimers were purified from B cell lines by affinity purification on L243 (HLA-DR) or SPVL3 (HLA-DQ). Peptide binding was evaluated with competitive ELISA using an automated workstation (7, 18–21). HLA heterodimers were incubated with biotinylated indicator peptide and serial dilutions of competitor peptide. As reference peptides, unlabeled forms of the biotinylated indicator peptide were used to assess validity in each experiment. The following reference sequences were used as labeled indicator peptides, and their IC_50_ values are indicated in parentheses: HA 306-318 (PKYVKQNTLKLAT) for DRB1*01:01 (2 nM), DRB1*04:01 (14 nM) and DRB1*11:01 (72 nM) and DRB5*01:02 (18 nM), YKL (AAYAAAKAAALAA) for DRB1*07:01 (5 nM); A3 152–166 (EAEQLRAYLDGTGVE) for DRB1*15:01 (41 nM), MT 2–16 (AKTIAYDEEARRGLE) for DRB1*03:01 (71 nM), B1 21–36 (TERVRLVTRHIYNREE) for DRB1*13:01 (46 nM), CTP 427–441 (VHGFYNPAVSRIVEA) for DRB1*09:01 (23 nM), TFR141–155 (TGTIKLLNENSYVPR) (360 nM) for DRB1*12:02, TFR607–620 (LNLDYERYNSQLLS) for DRB1*15:02 (4 nM). B7150–164 (LNEDLRSWTAADTAA) for DQ6 (DQA1*01:03/DQB1*06:03) (37 nM) and DQB45–57 (ADVEVYRAVTPLGPPD) for DQ8 (DQA1*03:01/DQB1*03:02; 98 nM).

After 24–72 h incubation (37°C), samples were neutralized with 50 μl 450 mM Tris HCl (pH 7.5), 0.3% BSA, 1 mM DM buffer and applied to 96-well MaxiSorp ELISA plates (Nunc) coated with 10 μg/ml L243. Bound biotinylated peptide was detected by streptavidin-alkaline phosphatase conjugate (GE Healthcare, Saclay, France). Emitted fluorescence was measured at 450 nm upon excitation at 365 nm. The peptide concentration preventing 50% binding of labeled peptide (IC_50_) was evaluated and data expressed as relative affinity: the ratio of the IC_50_ of test peptide to the IC_50_ of reference peptide. Mean ± SEM was calculated from two to three independent experiments. Relative affinities of 10 or less were considered high binders, and relative affinities of 10–100 were moderate binders (Tables II, III, Supplemental Table I).

### HLA transgenic mouse studies

This study used HLA class II transgenic mouse lines for the alleles HLA-DR1 (DRB1*0101), HLA-DR4 (DRB1*0401), HLA-DQ6 (DQB1*0602), and HLA-DQ8 (DQB1*0302), which were in each case maintained in the context of a homozygous knockout for murine H2-Aβ, as described previously (7, 18–20). Mice were maintained in individually ventilated cages and were used in experiments as age- and sex-matched, young adults. For CD4 T cell epitope mapping studies, mice were primed in one hind footpad with 25 μg Ag emulsified in Hunters Titermax Gold adjuvant (Sigma-Aldrich). At day 10, the draining popliteal lymph node was removed and disaggregated into a single-cell suspension for ELISPOT assays. The frequency of cells producing IFN-γ in response to Ag was quantified with ELISPOT (Diaclone; 2B Scientific, Oxon, U.K.) performed in HL-1 serum-free medium (BioWhittaker; Lonza, Slough, U.K.), supplemented with l-glutamine and penicillin–streptomycin (Life Technologies, Paisely, U.K.). Cells (2 × 10^5^) plus Ag were added to wells and plates and were incubated for 72 h at 37°C with 5% CO_2_. Unless otherwise indicated, peptide was added to wells at a final concentration of 25 μg/ml. Spots were counted on an automated ELISPOT reader (Autoimmun Diagnostika, Strasbourg, France). Response frequencies were expressed as ΔSFC/10^6^ cells, with the presence of an epitope being confirmed when the majority of mice in the immunized group responded with a magnitude greater than the mean number of spot-forming cells (SFCs) in medium only control + 2 SD. Mean + 2 SD background SFC for murine ELISPOT data are indicated in each case by a dotted line on the figures. The ELISPOT background ranges (per 10^6^ cells) were 0 to 30 SFC.

### Human cohorts and T cell assays

Leukocytes from healthy blood donors were collected (Blood Transfusion Center, Khon Kaen Hospital, Khon Kaen, Thailand). *B. pseudomallei* seropositive samples were selected on the basis of indirect hemagglutination assay titers ≥ 1:40 (22). HLA-DRB1 and DQB1 genotypes were determined with PCR sequence-specific primer (PCR-SSP) as previously described (23). PBMCs were isolated by Ficoll-Hypaque (Sigma-Aldrich) density gradient centrifugation and stored at −80°C until use. The frequency of T cell response to AhpC peptides was evaluated by IFNγ ELIspot (C.T.L.). Precoated 96-well polyvinylidene difluoride plates (MSIP; Millipore) were incubated overnight with 15 μg/ml IFN-γ Ab. One hundred microliter of cells at 2.5 × 10^6^ cells/ml, pulsed with 100 μl of 5 × 10^7^ CFU/ml of killed *B. pseudomallei* as positive control, with 50 μg/ml individual AhpC individual peptides or with medium alone as a negative control, were added in triplicate. Plates were cultured for 48 h, and IFN-γ secretion was detected by adding human IFN-γ detection solution for 2 h. IFN-γ–producing cells were quantified with Immunospot analyzer (C.T.L.). Results are expressed as mean SFC per 10^6^ cells of triplicates in the presence of Ag minus mean spots in the medium control (ΔSFC), and they are considered significant if values are above the mean medium-only control + 2 SD. The background range for these human ELISPOT data are shown in Fig. 2A (media only).

Blood was collected from 41 patients with culture-confirmed melioidosis at Sappasitthiprasong Hospital, Ubon Ratchathani, at a median of 5 d after admission to hospital (interquartile range 4–6 d). PBMCs were separated from heparinized blood by density centrifugation and counted with a Scepter handheld counter (Millipore) and cryopreserved at −80°C before transfer on dry ice to liquid nitrogen storage at Mahidol Oxford Tropical Medicine Research Unit. Cellular responses to 20 μg/ml AhpC protein, 1 μg/ml T cell epitope pool (Mabtech AB) and 2 μg/ml of a scrub typhus Ag r47 were measured with an 18-h IFN-γ ELISPOT assay using 96-well Multiscreen-I plates (Millipore) and a proprietary kit (Mabtech AB) according to the manufacturer’s instructions. To evaluate HLA class I and class II restriction of T cell responses to AhpC protein, additional ELISPOT assays were performed in the presence and absence of the anti-class I Ab (W6/32) and anti-class II Abs L243 (DR) and SPVL3 (DQ), used at a final concentration of 25 μg/ml. Plates were read using a CTL ELISPOT reader, and results were expressed as IFN-γ SFCs per 1 million PBMCs with subtraction of background responses in unstimulated control wells. Responses were defined as positive if values were greater than the mean of the medium only control + 2 SD and a minimum of 20 SFCs per 1 million PBMCs. Donor samples showing a highly nonspecific background response in the absence of Ag were not used. The median background ELISPOT measurement in SFCs (per 10^6^ cells) was 10 (range, 0–180), and there was no significant difference in background measurements between groups (by Mann–Whitney *U* test).

For intracellular cytokine staining with flow cytometry, PBMCs were cultured in the presence of AhpC protein (0.4 μg/well) or media only for 18 h. Brefeldin A (eBioscience) was added at 10 μg/ml. After 4 h of additional incubation, cells were stained for intracellular APC–IFN-γ, and cell surface markers (PerCP-antiCD3, FITC-antiCD4, and APC-H7-antiCD8; BD Biosciences). Samples were analyzed using a MACSQuant Analyzer 10 (Miltenyi Biotec) with Flowjo software.

## Results

### AhpC sequence contains strong HLA-DR and -DQ binding epitopes

In view of the fact that the *Salmonella typhi* hydroperoxide reductase is immunogenic and protective in experimental Salmonellosis (24) and that the *B. pseudomallei* homolog is seroreactive in clinical melioidosis (11), we set out to investigate the candidacy of AhpC (BPSL 2096) as a T cell Ag. A synthetic peptide panel of 20 mers overlapping by 10 aa was generated, covering the full coding sequence (Table I) and binding affinities determined for the HLA-DR alleles, HLA-DR1, -DR3, -DR4, -DR7, -DR9, -DR11, -DR13, -DR1501, and -DR1502 and the HLA-DQ alleles DQ6 (DQB1*0602) and DQ8 (DQB1*0302) (Table II, Supplemental Table I). Of the 17 peptides in the panel, all were able to bind some of the HLA alleles tested. One of the peptides, p6 (51–70), is noteworthy in that it binds virtually all the HLA-DR and DQ alleles tested at moderate to high affinity (Table III, Supplemental Table I).

**Table I.** AhpC BPSL2096 peptide panel (accession no. YP_108693.1)

Peptide	Peptide Name	AA Sequence
1	BPSL2096 (1–20)	MKTVGDKLEAFTVVAAKPGF
2	BPSL2096 (11–30)	FTVVAAKPGFNNHEENGQSA
3	BPSL2096 (21–40)	NNHEENGQSAFETVTEASFP
4	BPSL2096 (31–50)	FETVTEASFPGKWKIIYFYP
5	BPSL2096 (41–60)	GKWKIIYFYPKDFTFVCPTE
6	BPSL2096 (51–70)	KDFTFVCPTEIVEFAKLAKQ
7	BPSL2096 (61–80)	IVEFAKLAKQFEERDAVLLG
8	BPSL2096 (71–90)	FEERDAVLLGGSSDNEFVKL
9	BPSL2096 (81–100)	GSSDNEFVKLAWRREHKDLD
10	BPSL2096 (91–110)	AWRREHKDLDKLNHYSFGDV
11	BPSL2096 (101–120)	KLNHYSFGDVKGELIDQLGV
12	BPSL2096 (111–130)	KGELIDQLGVRDKEAGVALR
13	BPSL2096 (121–140)	RDKEAGVALRATFIVDPDNT
14	BPSL2096 (131–150)	ATFIVDPDNTIQHVSVNNLN
15	BPSL2096 (141–160)	IQHVSVNNLNVGRSPEEILR
16	BPSL2096 (151–170)	VGRSPEEILRILDGLQTDEL
17	BPSL2096 (161–182)	ILDGLQTDELCPCNRAIGGATL

**Table II.** Relative binding affinity of AhpC peptides to HLA-DR and -DQ molecules

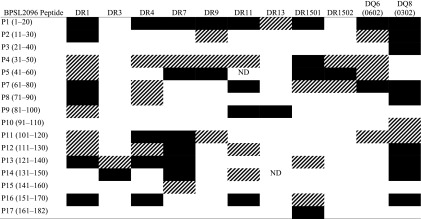

Results are derived from the relative binding ratio obtained by dividing the IC_50_ of each peptide by that of a reference peptide that binds strongly to the HLA molecule tested. Black-shaded squares indicate high-affinity binding. Diagonally hatched squares indicate moderate binding affinity. Unshaded squares indicate that no binding was detectable. Each peptide–MHC combination was evaluated in two independent experiments. Numerical values for these binding assays are given in Supplemental Table I.

ND, not done.

**Table III.** Relative binding affinity of wild-type peptide 6 and the altered variant of peptide 6 to HLA-DR heterodimers

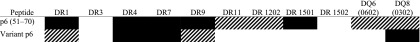

Results depict the relative binding ratio obtained by dividing the IC_50_ of each peptide by that of a reference peptide that binds strongly to the HLA molecule tested. Black-shaded squares indicate high-affinity binding. Diagonally hatched squares indicate moderate binding affinity. Unshaded squares indicate that no binding was detectable. Each peptide–MHC combination was evaluated in two independent experiments. Numerical values for these binding assays are given in Supplemental Table I.

BPSL2096 (51–70) p6, (KDFTFVCPTEIVEFAKLAKQ); BPSL2096 variant of p6, (KDFTFVCPTEIVEFAKQFEE).

### Immunization of HLA-DR and -DQ transgenic mice highlights HLA class II–determined immunodominant epitopes of AhpC

**FIGURE 1.**
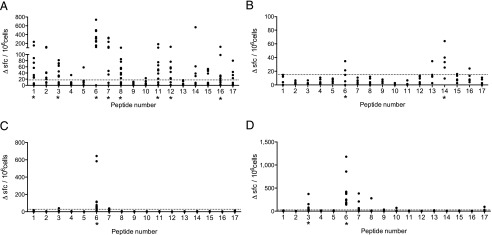
Immunization of HLA-DR and -DQ transgenic mice highlights HLA class II determined immunodominant epitopes of AhpC. Mice transgenic for HLA-DR1, *n* = 12, (**A**); HLA-DR4, *n* = 5 (**B**); HLA-DQ6 (DQB1*0602), *n* = 12 (**C**); and HLA-DQ8 (DQB1*0302), *n* = 12 (**D**) were primed with 25 μg rAhpC, and draining lymph node cells were assayed with IFN-γ ELISPOT in response to the indicated peptide at day 10. Data are plotted as SFCs per 10^6^ cells for individual mice. Responses to peptide were defined as positive if SFC > mean + 2 SD of the response in the absence of any Ag (shown as horizontal dotted line).

Binding studies of AhpC peptides to HLA-DR and -DQ heterodimers suggested a potentially complex pattern of peptide presentation to T cells. We immunized a panel of HLA-DR and -DQ transgenic mice with recombinant AhpC protein and determined the immunodominant T cell epitopes by IFN-γ ELISPOT assay in an in vitro recall response of lymph node T cells to the AhpC peptide panel (Fig. 1). Patterns of response determined by the different HLA heterodimers were distinctive: HLA-DR1 transgenics respond to epitopes in peptides 1, 3, 6, 7, 8, 11, 12, and 16; HLA-DR4 transgenics respond to epitopes in peptides 6 and 14. Of responses in the HLA-DQ transgenic lines, HLA-DQ6 mice showed a response that was large and entirely focused on p6, whereas HLA-DQ8 transgenics recognized p6 and p3. Thus p6 is identified as a promiscuous and immunodominant T cell epitope of AhpC, capable of stimulating a T cell response that encompasses presentation by diverse alleles of both HLA-DR and -DQ. A peptide-specific, Ag-dependent lymphocyte IFN-γ response is likely to be made by CD4 T cells. In defining CD4 versus CD8 responses, a starting point is that, in an HLA class II transgenic mouse—knocked out for murine class II and thus with a single, human class II molecule with which to restrict CD4 repertoire—any IFN-γ ELISPOT responses found to be Ag dependent would be most likely to be made by CD4 T cells; the case would seem strong considering that the peptide panel comprises 20 mers, which would be poorly accommodated in a class I peptide-binding groove. However, we found that a response to p6 (but not to staphylococcal enterotoxin B superantigen) was retained in HLA class II-negative, H2Aβ-knockout mice, most compatible with the notion of presentation through murine class I to CD8 cells and arguing that the response to p6 peptide in mouse comprises both a CD4 and CD8 component (see Supplemental Fig. 1).

### High-frequency T cell immune response to AhpC in exposed seropositive human donors from Khon Kaen, Thailand

**FIGURE 2.**
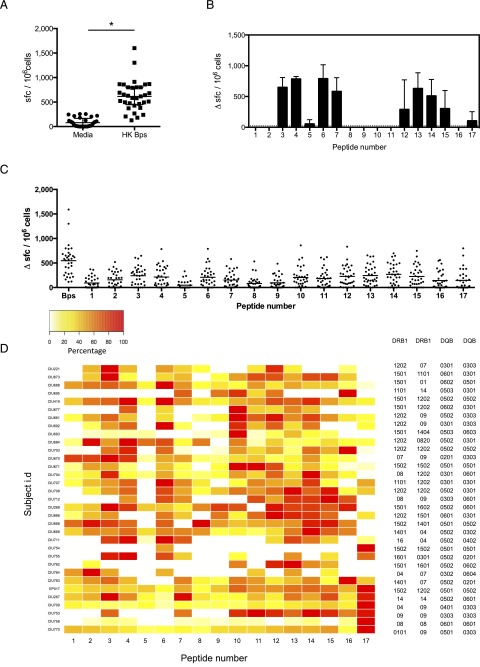
High-frequency T cell immune response in exposed seropositive human donors from Khon Kaen, Thailand. (**A**) T cell responses to heat-killed *B. pseudomallei* (HK Bps) in seropositive blood donors from the Khon Kaen region (*n* = 33). Statistical significance was determined using a Mann–Whitney *U* test. Median values with interquartile range for each group are marked. (**B**) T cell responses to the AhpC BPSL2096 peptide panel in a representative seropositive donor. Two SD above the mean of the media only control is shown as a horizontal dotted line. (**C**) Collated peptide T cell responses in the donor panel (*n* = 33). Mean values for responses to each peptide are marked as a horizontal line. Responses were defined as positive if SFCs > mean + 2 SD of the media-only control. (**D**) Heat map of percentile-ranked responses to each peptide in the donor panel and HLA genotype (*n* = 33). All responses for a given donor are expressed as a percentage of the biggest response measured for that donor. Higher responses are shown in red, and lower responses are shown in white. *p < 0.0001.

T cell ELISPOT responses to the AhpC peptide panel were analyzed in 33 *B. pseudomallei–*seropositive blood donors from the *B. pseudomallei–*endemic region of Khon Kaen in Thailand. All showed a positive T cell response to heat-killed *B. pseudomallei* (Fig. 2A). PBMCs from the donors were then analyzed with IFN-γ ELISPOT in response to each individual, overlapping AhpC BSPL2096 peptide. A representative donor response is shown in Fig. 2B; eight peptides induced high-frequency T cell responses in this donor, including p6. When responses from the full cohort of 33 seropositive donors is collated (Fig. 2C), it can be seen that at least some of the individuals in the cohort can respond to each of the peptides in the panel, sometimes at high frequencies of >700 SFC/10^6^. Given the binding data showing that many of these epitopes could be presented simultaneously by several HLA-DR and -DQ heterodimers in each individual, these large responses might not be surprising. The heat map representation of this dataset (Fig. 2D) shows that the sequence encompasses hotspots of common T cell recognition. Within this cohort, as would be anticipated in this geographical region, the most common alleles are DRB1*1202 (14 copies), DRB1*1501 (8 copies), and DRB1*0901 (8 copies). Of the 14 DRB1*1202^+^ donors, eight donors show a strong response to p10, making it possible that this heterodimer may present p10. Of the eight individuals who are DRB1*1501^+^, all show a strong response to p13.

### Survivors of acute melioidosis in Ubon Ratchathani have higher T cell responses to AhpC

**FIGURE 3.**
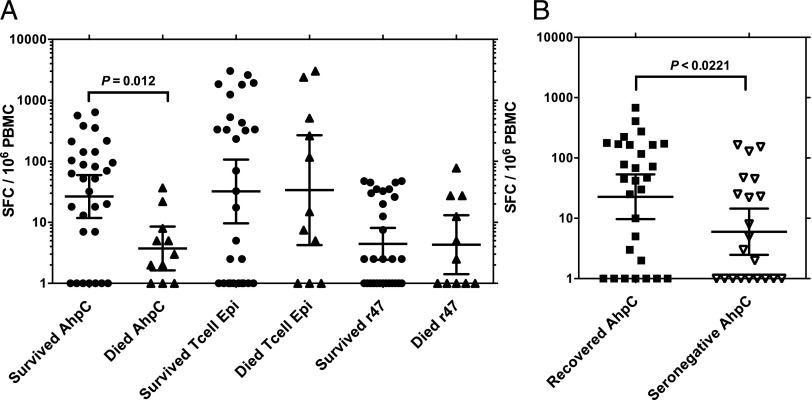
High T cell responses to AhpC protein are associated with survival in patients with acute melioidosis in Ubon Ratchathani. (**A**) Responses measured with 18 h ex vivo IFN-γ ELISPOT assay in PBMCs from patients with culture-confirmed melioidosis are shown for patients who survived (*n* = 30) versus those who died (*n* = 11). Responses were measured to 20 μg/ml AhpC protein, 1 μg/ml T cell epitope pool (“T-cell epi”) and 2 μg/ml of a scrub typhus Ag r47. (**B**) Recovered melioidosis patients have strong T cell responses to AhpC. Responses to 20 μg/ml AhpC protein measured with 18 h ex vivo IFN-γ ELISPOT assay in PBMCs are shown for patients 12 wk after culture-confirmed melioidosis (*n* = 30) compared with seronegative control subjects at the same hospital (*n* = 21). Error bars represent geometric means with 95% confidence intervals, and statistical significance was determined using the Mann–Whitney *U* test.

We then turned from analysis of T cell epitopes in healthy seropositive donors to analysis of T cell responses as a correlate of disease outcome in acute melioidosis patients. Higher responses to AhpC were seen in patients with acute melioidosis who survived the illness (arithmetic mean response, 113 SFCs per 10^6^ PBMCs) compared with patients who died (arithmetic mean response, 8 SFCs per 10^6^ PBMCs; *p* = 0.012; Fig. 3A). This relationship was not seen for responses to the T cell epitope pool or an irrelevant Ag (r47 from scrub typhus). The T cell IFN-γ production in response to AhpC protein was shown to be predominantly CD4 (Supplemental Fig. 2A, 2B) and HLA class II restricted by Ab blocking studies (Supplemental Fig. 2C). Patients who recovered from melioidosis also showed strong T cell responses to AhpC 12 wk later (arithmetic mean response, 103 SFCs per 10^6^ PBMCs), significantly higher than seronegative control subjects in the same region (arithmetic mean response, 30 SFCs per 10^6^ PBMCs; *p* = 0.0221; Fig. 3B). Interestingly, 8 of the 21 seronegative donors show a specific response to AhpC exceeding 20 SFCs per 10^6^ PBMCs. We interpret this to mean that, in this *Burkholderia*-endemic region, there are likely to be previously exposed individuals in whom specific Ab has waned faster than T cell memory has. We know that there can be divergence between exposure and seropostivity; in a larger Ubon cohort, only 74% of individuals who survive culture-confirmed melioidosis have IHA titers greater than 40, 12 weeks later (S. Dunachie, unpublished observations). An additional possibility is that there are epitopes within AhpC that are conserved between *B. pseudomallei* and other bacterial species.

### AhpC p6 epitope is partially deleted in a Cambodian clinical isolate, SR-039

**FIGURE 4.**
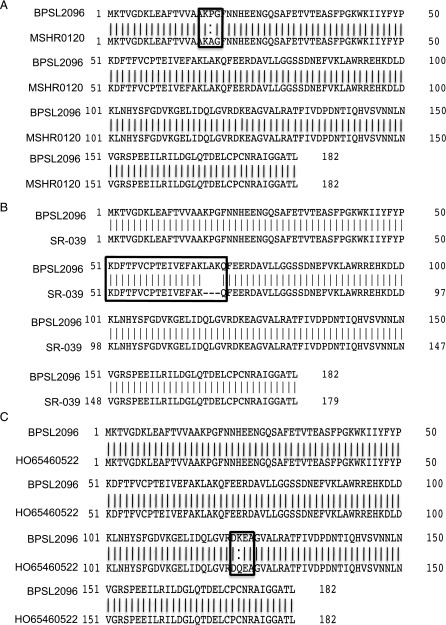
AhpC p6 epitope is partially deleted in a Cambodian clinical isolate, SR-039. Comparative analysis of AphC coding sequence reveals that AhpC is almost invariant. Alignment of the amino acid sequences for AhpC from K96243 (*top*) and SR-039 (*bottom*) is shown. Three variants of the AhpC alkyl hydroperoxide reductase protein were detected in three separate isolates; these included amino acid substitutions (**A**) Pro18Ala and (**C**) Lys123Gln and (**B**) a deletion of a tripeptide at positions 67–69 (Leu-Aln-Lys)—the p6 epitope region of the AhpC protein is indicated by the box in (B).

Since publication of the initial *B. pseudomallei* reference genome in 2004 (25), investigators in Asia, Africa, Australia, South America, and the Pacific have contributed clinical isolates and data for the sequencing and curation of >200 *B. pseudomallei* isolate genomes (http://www.sanger.ac.uk/resources/downloads/bacteria/burkholderia-pseudomallei.html#t_2). Comparative analysis of the AphC coding sequence revealed that AhpC is almost invariant. Three variants of the AhpC alkyl hydroperoxide reductase protein were detected in three separate isolates; these included deletion of a tripeptide at positions 67 to 69 (Leu-Aln-Lys; Fig. 4B) and amino acid substitutions Pro18Ala and Lys123Gln (Fig. 4A, 4C). In the case of the deletion, it results in a deletion within the p6 epitope. The strain to which this belongs was isolated in Siem Reap, Cambodia (accession number of sequence data ERS090378) from a pediatric case with a parotid abscess. However, the deletion was not seen in other isolates from the same region (M. Holden, unpublished data). Data from Cambodia indicates that *B. pseudomallei* is present in ∼30% of rice paddies in the Siem Reap region (26).

### Altered variant of peptide 6 can bind HLA class II heterodimers, but shows little or no T cell recognition

We synthesized the wild-type and altered variant peptide of p6 (i.e., the peptide that omits LAK and has the sequence KDFTFVCPTEIVEFAKQFEE) and measured their ability to bind HLA class II heterodimers. For all but one of the HLA alleles tested, we found that the variant peptide showed similar or lower affinity binding than wild-type p6 (Table III, Supplemental Table I), suggesting that this difference might influence immunity.

**FIGURE 5.**
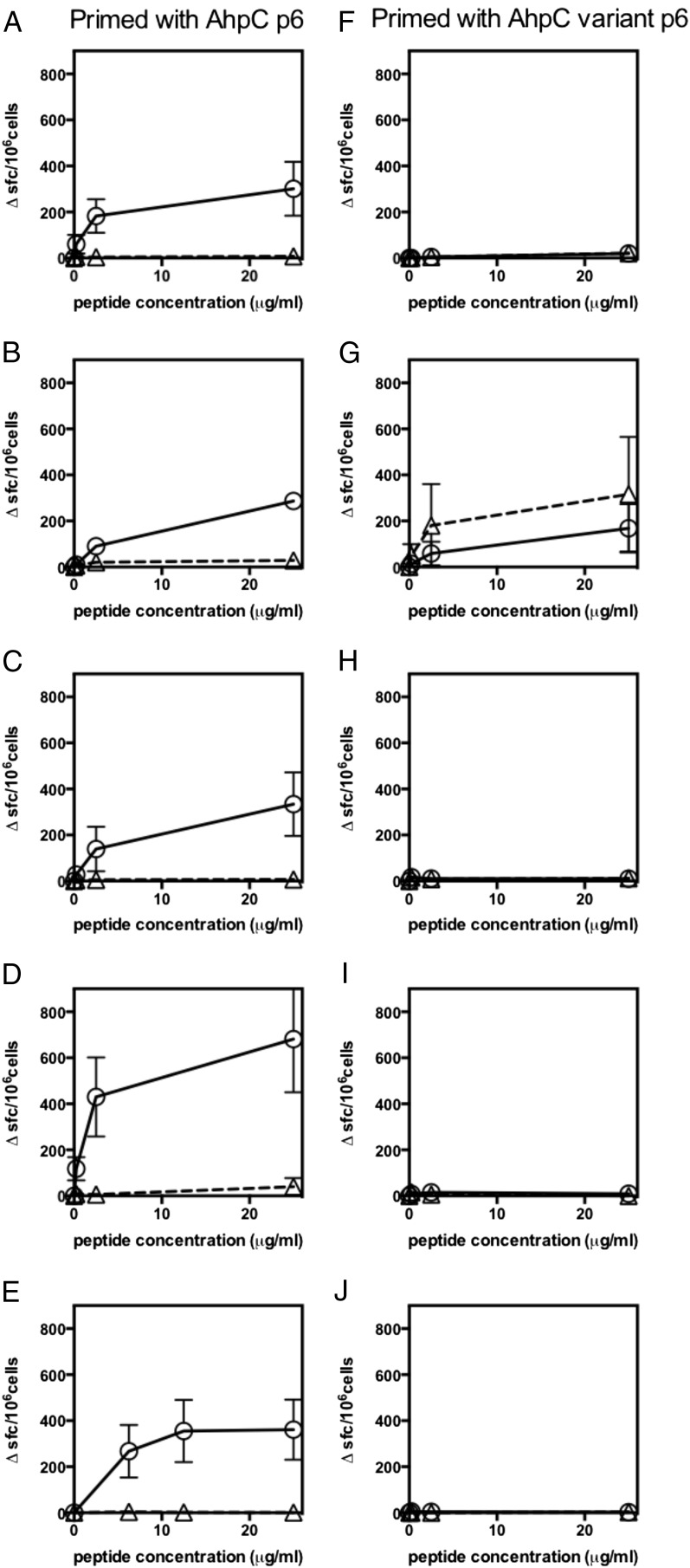
Responses of T cells from HLA class II transgenic mice to p6 or altered variant p6. (**A**) HLA transgenic mice (*n* = 3 per group) expressing the alleles (A) HLA-DR1, (**B**) -DR4, (**C**) -DQ8 (DQB1*0302), (**D**) -DR1502, (**E**) and -DR1501 were primed with 25 μg p6 peptide in adjuvant and draining lymph node cells assayed in triplicate by IFN-γ ELISPOT in response to the indicated peptide at day 10. They were assayed for responses to p6 (open circle) or altered variant p6 (open triangle). HLA transgenic mice (*n* = 3 per group) expressing the alleles (**F**) HLA-DR1, (**G**) -DR4, (**H**) -DQ8 (DQB1*0302), (**I**) -DR1502, and (**J**) -DR1501 were primed with 25 μg of altered variant p6 peptide in adjuvant, and draining lymph node cells were assayed in triplicate with IFN-γ ELISPOT in response to the indicated peptide at day 10. They were assayed for responses to p6 (open circle) or altered variant p6 (open triangle).

To test this suggestion, HLA transgenic mice were primed with p6 or altered variant p6. The response of draining lymph node cells to a titration of each peptide was then measured with INF-γ ELISPOT (Fig. 5). Across four HLA-DR and one HLA-DQ allele tested, DLN cells generated in response to p6 were unable to respond to the altered variant peptide (Fig. 5A–E). Furthermore, there was no secretion of the cytokines IL-4, IL-5, and IL-10 (data not shown). When mice were primed with the altered variant peptide, no T cell recognition was elicited in the HLA backgrounds tested, with the exception of a small response to both peptides after priming of HLA-DR4 mice with the altered variant peptide (Fig. 5F–I). Again, there was no secretion of the cytokines IL-4, IL-5, and IL-10 (data not shown).

## Discussion

There is a significant level of concern about a potential “time bomb” in Asia and elsewhere, because of the convergence of an increasing prevalence of diabetes (27) (the key risk-factor for melioidosis) and widespread exposure in the region to environmental *B. pseudomallei* (the causative agent for melioidosis). As such, there is a degree of overlap between the imperatives driven by public health in this region and by biodefense initiatives in the United States and elsewhere to identify plausible candidates for vaccine programs (28). In this work, we have studied AhpC as one such candidate identified as a serodominant Ag in an earlier study of *B. pseudomallei* immunogens targeted by the immune response of patients (11). Furthermore, it has long been appreciated that homologs of AhpC are highly immunogenic and protective in other bacterial infections (24, 28). The relationship between AhpC upregulation as part of the OxyR regulon, reducing the antibacterial effects of host-produced hydrogen peroxide and the induction of host adaptive immunity, was first demonstrated in *Salmonella typhimurium* infection (24). In this setting, macrophage expression of AhpC is upregulated, but the price paid for this upregulation is the induction of a strong CD4 T cell and Ab response. Similarly, AhpC is highly serodominant in immunoproteomic analysis of the *Bacillus anthracis* secretome, screened with sera from infected animals (29).

The annotated dataset of the *B. pseudomallei* transcriptome reported by Tan and colleagues shows BPSL2096 to be upregulated by oxidative stress or anaerobic conditions, as would be expected, but downregulated in the context of chronic lung infection (30). This report highlights the trade-off for an intracellular bacterial pathogen, needing to evade innate oxidative stress by upregulation of peroxidases, yet needing to keep expression low to evade recognition by adaptive immunity. A key message from the current study was the extent to which, whether assessed in Thai seropositive human donors or in the more reductionist setting of HLA transgenic mice, T cell responses are high frequency, encompass a large number of epitopes across the protein sequence, and have the ability to bind strongly to diverse HLA heterodimers of different isotypes. There is evidence supporting a role of mammalian peroxiredoxins in danger-associated molecular patterns activation: secreted peroxiredoxin family proteins upregulated in the brain after stroke act as inducers of IL-23 from infiltrating macrophages through TLR2 and TLR4 (31). That related family members can act as danger-associated molecular patterns raises the interesting possibility that, like bacterial flagellin Ags, the AhpC family members may conceivably achieve strong immunogenicity through stimulation of both adaptive immunity and TLRs, thus being functionally self-adjuvanted.

A noteworthy aspect of the findings in our study was the strong response around the p6 region of AhpC and the fact that immunogenicity of this region is lost in one of the archived Cambodian isolates. Furthermore, the p5/p6 region of immunogenicity overlies the active site of the hydroperoxide reductase enzymatic active site (32). Could this support a ‘Red Queen’ hypothesis (33) such that T cell immunity has targeted a region for which the price of mutation is disadvantageous to bacterial function? It would be difficult to make a strong case for host immunity–driven selection pressure forcing variation in the p6 region of the sequence; human-to-human transmission of *B. pseudomallei* is described only rarely because it is not an obligate human pathogen and, as shown in this study and in our earlier studies, there is an abundance of T cell epitopes targeted by the immune response. On the other hand, recent data suggest the potential for significant longitudinal sequence variation in vivo, whether driven by antimicrobial therapy or host immunity (34, 35). We considered the possibility that the Cambodian p6 variant sequence may be an altered peptide ligand—that is, with the ability to signal a deviated rather than a null response phenotype. However, we found no evidence to suggest that any of the cytokine responses for which we screened could be elicited in response to this sequence. The fact that responses to the variant sequence seem to be absent, but with the exception of a clear T cell response observed in the context of presentation by HLA-DR4, emphasizes the point that the immune repertoire to bacterial Ags, as to all Ags, can be exquisitely sensitive to host immunogenetic variation.

An enduring conundrum in studies of *B. pseudomallei* immunity has been the simple matter of focus on the key determinants of host protection. The fact that immune dysregulation associated with diabetes or alcohol misuse is a significant risk factor for melioidosis—yet more explicit disruption of CD4 immunity such as HIV infection appears less important—has supported a notion that host defense may be largely innate (36). This study identified eight melioidosis patients coinfected with HIV and found no explicit change in disease severity or outcome, although this is likely too small a dataset to draw firm conclusions (36). On the other hand, murine infection studies emphasize the importance of classical, CD4 IFN-γ bacterial immunity (8). The present study offers two important additions to this discussion. First, human seropositive donors who are healthy and thus presumably keeping at bay any progression to clinical melioidosis, despite living in an endemic region, show high frequency cumulative T cell responses to AhpC. Second, we found that among patients with acute melioidosis, survival was associated with a strong class II–restricted T cell response to AhpC, a highly immunodominant protein Ag, mapped both in the human and mouse studies. These pieces of evidence are in line with the expectation that T cell control of *B. pseudomallei* infection is indeed probably not dissimilar to the central role of T cell control in other intracellular bacterial infections such as *Salmonella* and tuberculosis (37, 38).

## Supplementary Material

Data Supplement
