# Supplementary material for: T Cell Immunity to the Alkyl Hydroperoxide Reductase of Burkholderia pseudomallei: A Correlate of Disease Outcome in Acute Melioidosis
Source: J Immunol. 2015 Apr 10;194(10):4814–24. doi: 10.4049/jimmunol.1402862 (PMC4416739; doi:10.4049/jimmunol.1402862)
Supplement: Data Supplement [file JI_1402862.zip › JI_1402862_Supplemental_Material_1.pdf]

Supplementary Table I. Relative binding affinity of AhpC peptides to HLA-DR and -DQ molecules.

BPSL2096 sequences	DR1	DR3	DR4	DR7	DR9	DR11	DR13	DR1501	DR1502	DQ6 602	DQ8 302
P1 [1-20]	**5**	>195	**1**	**0.8**	**10**	**3**	**94**	**12**	1 562	**3**	**2**
P2 [11-30]	**15**	>195	114	>358	**97**	217	>135	>184	>5 020	**100**	**17**
P3 [21-40]	>2 697	>195	>140	126	110	>247	>135	>184	>5 020	>222	**1**
P4 [31-50]	**34**	>195	**65**	**64**	**22**	**50**	>135	**1**	**70**	**84**	>625
P5 [41-60]	**34**	>195	>140	**3**	**4**	ND	>135	**1**	**5**	**29**	>625
P7 [61-80]	**11**	>195	**86**	120	103	**1**	>135	**69**	**95**	**11**	**16**
P8 [71-90]	**17**	>195	**93**	230	152	141	>135	>184	>5 020	179	**19**
P9 [81-100]	**54**	>195	>140	320	540	**1**	**7**	>184	3 742	>222	>625
P10 [91-110]	>2 697	>195	>140	360	548	115	>135	>184	3 000	>222	**32**
P11 [101-120]	**24**	167	**7**	**0.7**	**23**	>247	>135	148	563	**24**	**33**
P12 [111-130]	**66**	>195	**52**	**19**	107	**82**	>135	>184	2 175	105	**7**
P13 [121-140]	**2**	**24**	**10**	**8**	355	>247	>135	**23**	1 151	158	**2**
P14 [131-150]	1 000	**3**	>140	**2**	204	**100**	ND	>184	300	>222	**19**
P15 [141-160]	>2 697	>195	>140	**64**	171	158	>135	>184	>5 020	>222	>625
P16 [151-170]	**18**	>195	20	>358	447	20	>135	**37**	2 000	>222	**16**
P17 [161-182]	1 000	>195	>140	179	371	144	>135	**14**	1 035	>222	625
P6 [51 – 70]	**9**	>119	**9**	**0.3**	**18**	**43**	ND	**7**	222	**80**	**26**
Variant P6 (KDFTFVCPTEIVEFAKQFEE)	**50**	>119	**20**	**1**	**21**	201	ND	176	>3333	>143	**1**

*Results are expressed as a relative binding ratio obtained by dividing the IC_{50} of each peptide by that of a reference peptide that binds strongly to the HLA molecule tested. Lower numbers correspond to a higher binding affinity. Numbers in red (ratio of 20 or less) = high affinity binding; numbers in green (ratio 20-100) = moderate binding affinity. Each peptide - MHC combination was evaluated in 2 independent experiments. ND, not done.

Supplementary Figure 1

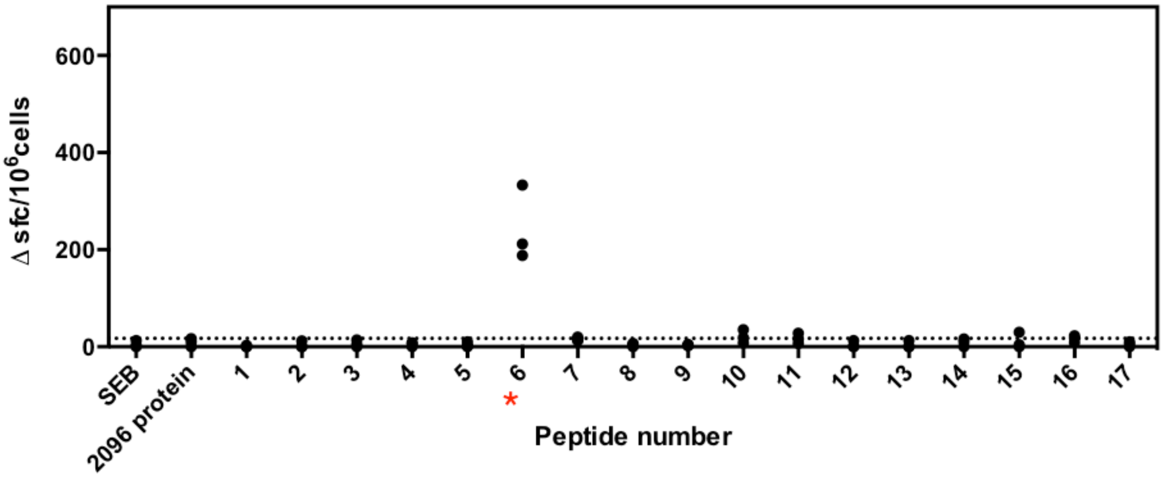

Supplementary Figure 1. Response of H2Aβ-/- mice to the AhpC peptide panel. Three male mice were primed in one hind footpad with 25µg AhpC/ Titermax and DLN cells assayed at d10 for IFNγ ELIspot responses to individual peptides, to whole recombinant AhpC protein or to SEB (as positive control for class II-presented CD4 responses). A dotted line indicates mean medium control + 2SD. A positive peptide response is indicated by an asterisk.

Supplementary Figure 2

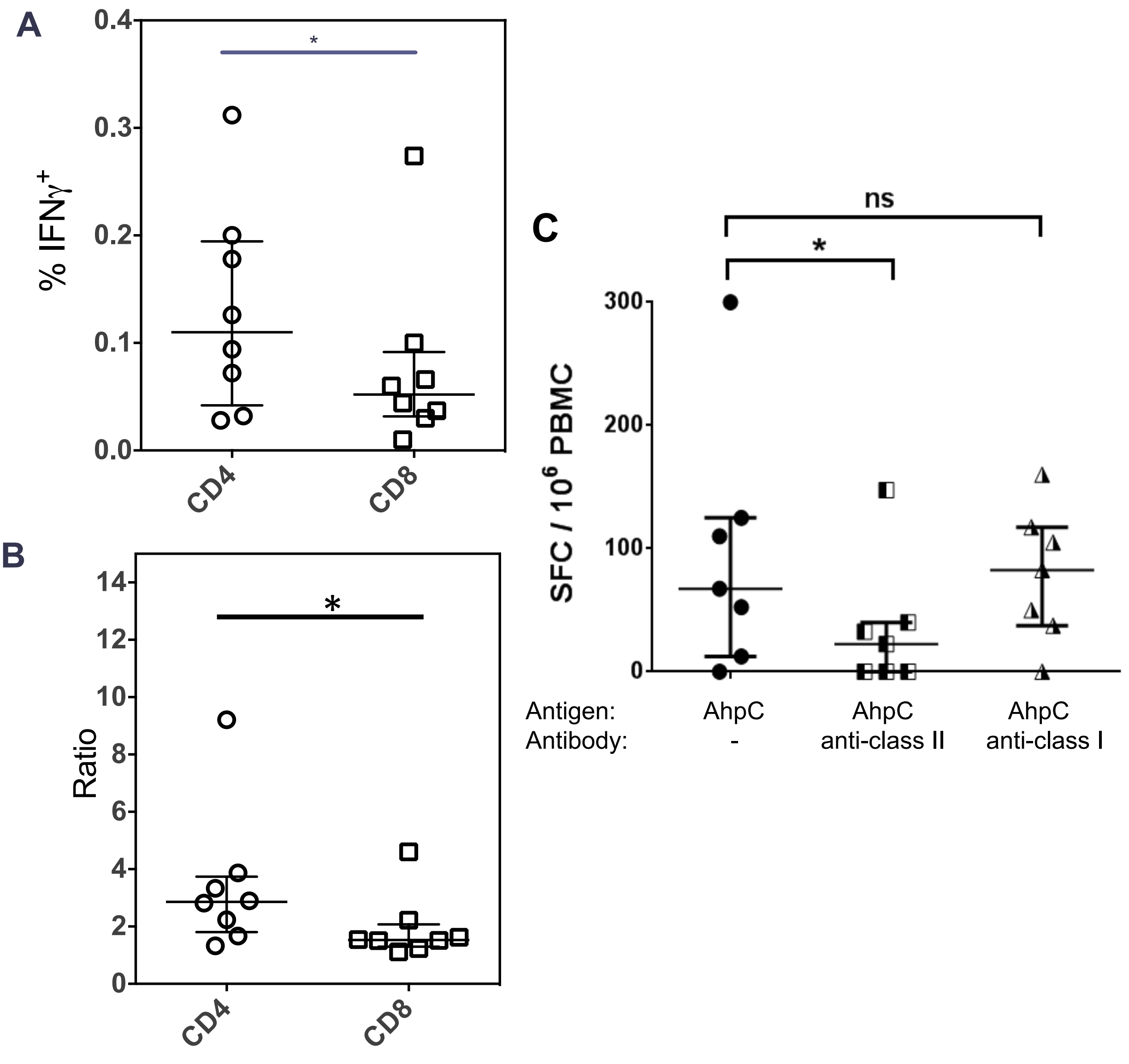

Supplementary Figure 2. CD4 and CD8 characterization of melioidosis patient responses to AhpC by intracellular cytokine staining (ICCS) and ELIspot. PBMC from 8 donors were cultured in the presence of AhpC protein (0.4µg/well) or media only for 18h. (A) Percent of antigen-specific IFNγ secreting cells, back-gated for CD4 and CD8. The median % IFNγ secreting CD4 cells after AhpC stimulation for 18 hours was 0.11%, compared to 0.052 % for CD8 cells (n=8 subjects from Melioid Cohort Week 12). t-test Wilcoxon, *indicates *P*=0.0078. (B) Ratio of IFNγ secreting cells (stimulated/unstimulated) for cells back-gated on CD4 and CD8. The ratio of IFNγ secreting CD4 cells from AhpC stimulated to unstimulated PBMC for 18 hours was 2.9 compared while the ratio from CD8 was 1.5 (n=8 subjects from Melioid Cohort Week 12). t-test Wilcoxon, *indicates *P*=0.0078. (C) ELIspot responses to AhpC were measured in the presence or absence of mAbs against HLA class II (L243 and SPVL3) and HLA class I (W6/32). *indicates *P*=0.0313; ns, not significant. Data analysis by Wilcoxon.
